# The resting membrane potential of hSC-CM in a syncytium is more hyperpolarised than that of isolated cells

**DOI:** 10.1080/19336950.2021.1871815

**Published:** 2021-01-19

**Authors:** Dieter V. Van de Sande, Ivan Kopljar, Alaerts Maaike, Ard Teisman, David J. Gallacher, Loeys Bart, Dirk J. Snyders, Luc Leybaert, Hua Rong Lu, Alain J. Labro

**Affiliations:** a Department of Biomedical Sciences, University of Antwerp, Antwerp, Belgium; b Global Safety Pharmacology, Non-Clinical Safety, Janssen R&D, Beerse, Belgium; c Centre of Medical Genetics, University of Antwerp, Antwerp, Belgium; d Department of Basic and Applied Medical Sciences, Ghent University, Ghent, Belgium

**Keywords:** electrophysiology, Whole cell patch-clamp, input resistance, Kir2.1, action potential

## Abstract

Human-induced pluripotent stem cell (hiPSC) and stem cell (hSC) derived cardiomyocytes (CM) are gaining popularity as in vitro model for cardiology and pharmacology studies. A remaining flaw of these cells, as shown by single-cell electrophysiological characterization, is a more depolarized resting membrane potential (RMP) compared to native CM. Most reports attribute this to a lower expression of the Kir2.1 potassium channel that generates the I_K1_ current. However, most RMP recordings are obtained from isolated hSC/hiPSC-CMs whereas in a more native setting these cells are interconnected with neighboring cells by connexin-based gap junctions, forming a syncytium. Hereby, these cells are electrically connected and the total pool of I_K1_ increases. Therefore, the input resistance (Ri) of interconnected cells is lower than that of isolated cells. During patch clamp experiments pipettes need to be well attached or sealed to the cell, which is reflected in the seal resistance (Rs), because a nonspecific ionic current can leak through this pipette-cell contact or seal and balance out small currents within the cell such as I_K1_. By recording the action potential of isolated hSC-CMs and that of hSC-CMs cultured in small monolayers, we show that the RMP of hSC-CMs in monolayer is approximately −20 mV more hyperpolarized compared to isolated cells. Accordingly, adding carbenoxolone, a connexin channel blocker, isolates the cell that is patch clamped from its neighboring cells of the monolayer and depolarizes the RMP. The presented data show that the recorded RMP of hSC-CMs in a syncytium is more negative than that determined from isolated hSC/hiPSC-CMs, most likely because the active pool of Kir2.1 channels increased.

## Introduction

**Figure 1.**
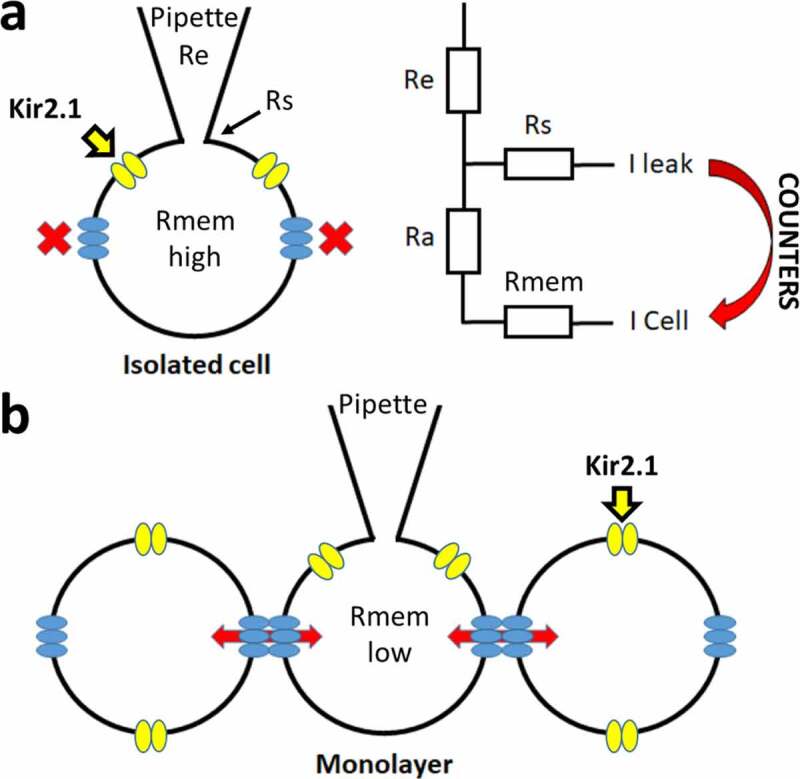
Schematic representation of the patch clamp technique and corresponding resistances. (a) Simplified overview of the patch clamp pipette attachment to the isolated cell (ruptured whole-cell configuration) is shown. Kir2.1 channels are represented in yellow and in blue the hemi-channels with no connection to other cells, leading to a high Rmem. A circuit diagram of the different resistances is shown on the right with Re, Rs, Ra and Rmem representing the electrode (pipette), seal, access and cell (or membrane) resistances, respectively. The leak current (I-leak), originating from the Rs, can counter the currents of the cell (I-cell). (b) Overview of patching a cell in a monolayer whereby cells are connected by gap junction channels (composed of two hemichannels from neighboring cells). The electrical and chemical connectivity between cells is indicated by the red arrows, which leads to a lower Rmem compared to isolated cells (panel a)

*In vitro* derived cardiomyocytes from human stem cells (hSC-CM) or induced pluripotent stem cells (hiPSC-CM) are emerging models for both disease modeling and pharmacology. As the cells are derived from a human source, hiPSC-CM and hSC-CM contain a repertoire of proteins resembling native cardiac myocytes [1,2]. These cells are being used in a multitude of applications, one of which are drug safety studies to detect adverse cardiac side effects [3]. In these settings, high-throughput assays are used such as calcium imaging, microelectrode array (MEA) and impedance measurements, performed on cell monolayers [4,5]. Presently, hiPSC-CMs are still not fully comparable to native cardiomyocytes with a frequently reported drawback that the resting membrane potential (RMP) is depolarized over 20 mV. An important current for setting a stable RMP is the I_K1_ K^+^ current, generated by the Kir2.1 (KCNJ2) channel [6,7], and the expression of this current has been reported to be lower in hiPSC-CMs [8–15]. Most of these findings were obtained from measuring isolated hiPSC-CMs and recent studies show that the RMP and action potential (AP) waveforms of hiPSC-CM in 3D structure tissue resemble its native counterpart more closely. In these 3D cultures the I_K1_ current appeared sufficiently expressed, hinting toward the idea that a technical issue could contribute to the more depolarized RMP in patch clamp measurements of isolated cells [16]. A factor that could play a role, as shown by *in silico* modeling, is the balance between the seal resistance of the patch (Rs) and the input resistance (Ri) of the cell [17]. Rs indicates the quality of the connection between the patch-pipette and the cell. If this connection is not tight, this results in a lower Rs value. Consequently, ions will leak through this connection yielding a leak current that generally has a reversal potential of 0 mV. If too large, this leak current counters small repolarizing currents within the cell such as the I_K1_ current, resulting in a depolarization of the RMP [17]. As hiPSC-CMs are reported to be significantly smaller in size compared to native cardiomyocytes [16], their ratio of repolarizing I_K1_ versus leak through Rs will probably be smaller than that of native CMs [18]. This ratio should also be lower in an isolated cell compared to that of a cell patched in a monolayer or 3D culture, since the latter is connected to its neighboring cells by connexins-formed gap junctions increasing the total I_K1_ current amplitude [19,20] (Figure 1). To study this, we determined the RMP and AP waveform of isolated hSC-CMs and of hSC-CMs in a monolayer (i.e. hSC-CM connected to each other). To strengthen that the ratio between I_K1_ and leak through Rs affects the RMP, CHO cells were transiently transfected with Kir2.1 as this results in cells with a variable I_K1_ expression. The results indicate that the leak currents caused by a low Rs value can counter the I_K1_ current depolarizing the RMP. Interestingly, the RMP value in hSC-CMs in monolayer (i.e. forming a gap junction coupled syncytium) was significantly more hyperpolarized than that of isolated cells, and chemically isolating the cells in the monolayer by adding carbenoxolone consequently depolarized the RMP.

## Material and methods

### Cell culture of hSC-CM and CHO cells

hSC-CMs (Cor.4 U) were acquired from Ncardia (Cologne, Germany) as nitrogen frozen vials containing 250 K cells (Ax-B-HC02-MPC) and brought into culture following the instructions of the provider in their hSC-CM media. Cells were incubated in a Galaxy B incubator (RS Biotech, Irvin, United Kingdom) at 37°C and 5% CO_2_, seeded on fibronectin-coated coverslips in a 24-well plate. Cells were cultured for a maximum period of two weeks and culture medium was refreshed every 2days. The fibronectin coating was done by adding 400 µl of a 10 µg/ml fibronectin solution in PBS with Ca^2+^ and Mg^2+^  (Sigma-Aldrich, St.Louis, MO, USA) to the well with the coverslip for 3 hours at 37°C. Only one batch of differentiated hSC-CMs was used to avoid inter batch variability (LOT°: CB1091CL_V_250 K). hSC-CMs were seeded at a density where monolayers were formed, but also isolated cells were present at the sides. Monolayers were defined as a group of more than 10 connected cells, with the patched cell in the center of the layer.

CHO cells were incubated in F12-HAM nut mix media supplied with 10% FBS and 1% penicillin/streptomycin (Gibco – Thermo Fisher, Waltham, MA, USA) at 37°C and 5% CO_2_ in 35 mm plastic dishes (VWR, Radnor, PA, USA). Transfection of 2.5 to 5 µg of human Kir2.1 cDNA (DNASU, Tempe, AZ, USA [21]), cloned in pCDNA3 vector, was performed 24 hours prior to patch clamp measurements, at a cell confluence of 50–60%, using a transient transfection with lipo2000 reagent (Invitrogen, Carlsbad, CA, USA). 0.5 µg eGFP (peGFP-vector) was co-transfected to visualize successfully transfected cells during patch clamp experiments. For patch clamp measurements, CHO cells were dissociated from the culture dish by a brief 0.5% trypsin treatment and subsequently transferred to the recording chamber of the patch clamp setup. To visualize the cells for patching an Eclipse TE2000-U-inverted fluorescence microscope was used (Nikon instruments Europe, Amsterdam, Netherlands).

### hSC-CM current clamp and CHO Kir2.1 voltage clamp experiments

AP waveforms from the hSC-CMs are recorded using the current clamp mode of the Axopatch 200B amplifier (Axon CNS Molecular devices, San Jose, CA, USA) and currents were digitized using a Digidata 1440A (Axon CNS Molecular devices). Intracellular solution (ICS) is backfilled in 1.2 mm quick-fill borosilicate glass pipettes (World Precision Instruments, Sarasota, FL, USA) with a resistance of 3–5 MΩ pulled using a horizontal P-2000 puller (Sutter Instrument Co., Novato, CA, USA) and subsequently heat polished. ICS consists of (in mM): KCl 150, NaCl 5, CaCl_2_ 2, MgCl_2_ 5, EGTA 5, HEPES 10 and adjusted to pH 7.2 with KOH. Perforation of the cell membrane is achieved by supplementation of the ICS with 0.84 mM amphotericin B (A9528, Sigma-Aldrich). Coverslips with hSC-CMs were transferred to the recording chamber of the patch clamp setup and superfused continuously at 1 ml/min with an extracellular solution (ECS) composed of (in mM): NaCl 150, KCl 5.4, CaCl_2_ 1.8, MgCl_2_ 1, glucose 15, HEPES 15, Na-pyruvate 1, and adjusted to pH 7.4 with NaOH. When the filled patch pipette made contact with the ECS, junction potentials were compensated before sealing of the cell. On average a seal resistance (Rs) of 1–2 GΩ was achieved and hSC-CM cells with a Rs lower than 1 GΩ were discarded from analysis. Prior to gaining access to the cell by perforation, leak currents were monitored at −80 mV for Rs calculations. After obtaining an adequate access, AP waveforms were recorded in the current clamp mode. Monolayers and isolated cells were not paced and spontaneous APs [22] were recorded over 10 seconds per sweep. Due to the spontaneous beating of the hSC-CMs in monolayers, it was not possible to do voltage clamp experiments because of continuous input from neighboring cells.

I_K1_ current recordings in CHO cells were performed using the whole-cell patch clamp technique in voltage clamp mode. ICS consisted of (in mM): KCl 110, MgCl_2_ 2, HEPES 10, K_2_ATP 5, K_4_BAPTA 5 and adjusted to pH 7.2 with KOH. The ECS contained (in mM): NaCl 145, KCl 4, CaCl_2_ 1.8, MgCl_2_ 1, glucose 10, HEPES 10, and adjusted to pH 7.4 with NaOH. Pipettes were horizontally laser pulled with a resistance of 1–2 MΩ. Prior to patching the cell, a compensation for junction potentials was performed followed by sealing of the cell to a resistance of 1–2 GΩ. Cells with an Rs lower than 1 GΩ were discarded from analysis, except mentioned otherwise. After whole cell access was achieved, the cell capacitance and access resistance (Ra) was compensated for 80%. Cells with a voltage error >5 mV, after compensation, were discarded during analysis. Cells were clamped at a holding potential of −80 mV before conducting voltage clamp experiments. The voltage clamp protocol for I_K1_ recordings is represented in. Currents were sampled at 10 kHz after passing a 5 kHz low-pass filter. Current clamp recordings were made on the same cell, using the same protocol used for AP recordings, to determine the cell’s RMP.

### Incubation of hemichannel blockers, and acute addition of gap junction blocker Carbenoxolone

Gap-26 or Gap-27 (Tocris Bioscience, Bristol, UK) are peptides that block hemichannels and with some delay also gap junctions; sequences designed for Cx43 also inhibit other connexin isotypes [23–25]. These peptides were added to the cell culture medium (200 µM) and isolated hSC-CMs were incubated for 3 hours at 37°C, 5% CO_2_. Afterward the coverslip was transferred to the chamber of the patch clamp setup in ECS supplemented with Gap-26 or Gap-27 (200 µM). Recordings were performed within the hour. The electrophysiological parameters of isolated hSC-CMs exposed to the Gap peptides were compared to control cells that followed the same incubation protocol but without addition of Gap peptides.

Carbenoxolone (Cbx) (Sigma-Aldrich, St. Louis, MO, USA) was added during the patch clamp experiment using a custom build pressurized fast perfusion system. Cbx was dissolved in ECS at concentrations of 100 and 200 µM. Cbx was added when performing experiments on hSC-CMs in monolayer to chemically and electrically isolate the recorded cell from its neighboring cells by blocking the gap junction channels. After obtaining whole cell access to an hSC-CM in a monolayer, Cbx (100 and 200 µM) was washed in for 5 minutes and APs were recorded continuously in current clamp mode (data not shown).

### Data analysis and statistics

**Figure 2.**
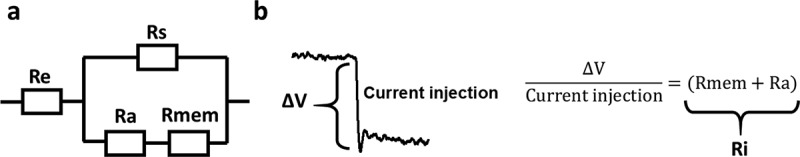
Methodology of Ri calculation. (a) Circuit diagram of the resistances in the patched cell configuration. Electrode resistance (Re) stands in series with the patched cell, whereby the Rs stands in parallel with the Ra and the Rmem, which together form the input resistance Ri. (b) Ri can be calculated, in current clamp mode, from the difference in potential (ΔV) after a current injection (−0.05 nA injected), a representative current recording is shown on the left. Using Ohm’s law and taking into account the circuit represented in panel a, Ri can be calculated as shown on the right

Current and AP data were analyzed using the pClamp10 software (Axon CNS Molecular devices). For AP waveform analysis, five APs were analyzed per cell, to avoid beat to beat variability, and the mean was calculated for every parameter. The following parameters were analyzed: peak, amplitude, maximal diastolic potential (MDP), upstroke velocity (dV/dTmax), AP duration at 90% of repolarization (APD90) and RMP. RMP values were obtained prior to AP depolarization. Upstroke velocity is calculated by using the first differential on the depolarization part of the AP waveform, from which the maximal velocity can be determined. Represented values are the MEAN ± SEM with n the number of cells analyzed. Rs was determined before obtaining cell access by applying a −10 mV step voltage protocol (voltage clamp configuration) whereby the corresponding current amplitude is a measure for the leak through the seal (i.e. connection between patch pipette and cell), which is used to calculate Rs with Ohm’s law. To obtain an adequate access to the cell, we waited approximately three minutes for the amphotericin B to perforate the cell membrane. The input resistance (Ri), which is proportional to the net currents present in the cell, was determined in current clamp mode from the change in membrane potential upon −0.05 nA current injection for 2 ms in duration. Note that a very small fraction of the current injection may pass the Rs as it is a parallel circuit (Figure 2a). However, with Rs values larger than 1 GΩ the fraction of current that leaks through the seal is negligible. It has to be noted that in this Ri calculation there is a minor contribution of the access resistance (Ra) but since the concentration of amphotericin B was the same in each patch clamp experiment no major variability in Ra is expected between the analyzed hSC-CM cells (Figure 2). For Kir2.1 expressing CHO cells no amphotericin B was added to the ICS as the whole-cell configuration was achieved by rupturing the membrane patch by applying suction pulses. Therefore, contribution of Ra was even lower as access is better in ruptured patch configuration compared to perforated patch. Electrode resistance (Re) was the same for all recordings and therefore a constant in the experiment. The ratio Rs/Ri was calculated using the obtained Rs and Ri values.

I_K1_ current was analyzed by normalizing the maximum current amplitude at each potential to the cell capacitance (pA/pF), without performing a leak subtraction beforehand. Values are given as MEAN ± SEM with n the number of cells analyzed. Plotting these current densities as a function of the applied potential yielded the current density-voltage (IV) plots. Ri and I_K1_ density were compared to highlight that an increase in I_K1_ density was reflected in a decrease in Ri.

Sigmaplot 11 software (Systat software inc., Chicago, IL, USA) was used for statistical analysis of the data and the generation of the figures. For statistical testing, the unpaired student t-test was performed while the statistical analysis for the wash in of Cbx on hSC-CMS monolayer cells was performed with a paired student t-test. Significance was obtained if the p-value was <0.05.

## Results

**Figure 3.**
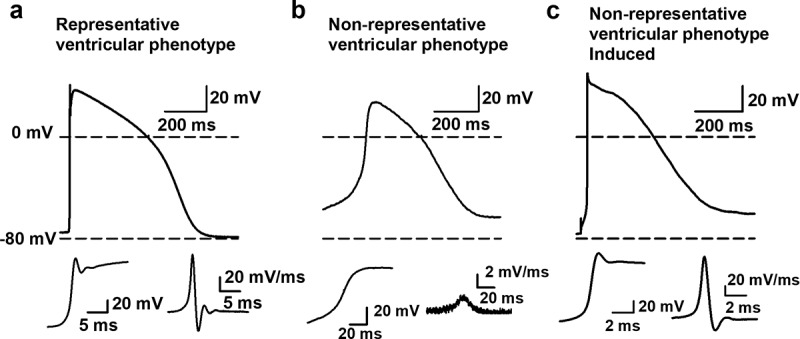
Representative and non-representative ventricular AP phenotype. (a) An example AP waveform of a cell with representative ventricular phenotype where the RMP is close to −80 mV and a fast upstroke velocity is observed. The insets at the bottom, on the left a magnification of the depolarization phase of the AP is shown with on the right the first differential of this depolarization phase that represents the dV/dtmax of the AP. (b) An example of a cell with a non-representative ventricular phenotype is represented, displaying a RMP more depolarized than −80 mV and a slow upstroke velocity. Magnification of the depolarization phase and the first differential of it are shown below. (c) Same cell as represented in panel b but now displaying the elicited AP upon a negative current injection (−0.05 nA). By the negative current injection the RMP was brought toward −80 mV. Subsequent induction of an AP leads to a fast upstroke velocity, which indicates that the cell is not a nodal type hSC-CM but a ventricular type cell with a more depolarized RMP

The RMP and spontaneous APs of isolated hSC-CMs and of hSC-CMs in a monolayer were recorded using the perforated patch clamp technique. Of note is that the isolated and monolayer cells were from the same batch to exclude differences attributed to batch-to-batch variability in hSC-CM differentiation. All APs were recorded and quality was assessed by comparison with native ventricular AP data. Cells were categorized as representative ventricular phenotypes when the RMP was more hyperpolarised than −60 mV and the AP amplitude was at least 80 mV, as the amplitude of a physiological ventricular CM is around 100 mV [26]. During depolarization this amplitude needs to be reached within a couple of milliseconds, therefore an upstroke velocity above 40 mV/ms was taken as third cutoff value, taking into account the temperature. In native human CM the upstroke velocity is substantially faster, around 200–250 mV/ms [26,27], but our measurements were done at room temperature (21°C ± 1°C). Therefore, the reported upstroke velocities are an underestimation as the Nav1.5 generated sodium current is temperature sensitive and activates faster at higher temperatures [28,29]. The representative ventricular phenotype cells also displayed a clear peak and dome characteristic (Figure 3a), which is also observed in native human ventricular CMs. In contrast, the non-representative ventricular phenotype cells had a more depolarized RMP, often displaying a slower upstroke velocity (below 40 mV/ms). Consequently, non-representative ventricular phenotype cells showed a more nodal-like waveform (Figure 3b). However, we recorded spontaneous APs and to exclude that nodal cells were included in the data set we secured that all non-representative ventricular phenotype cells could be converted to ventricular type APs by injection of a negative current (around −0.05 nA) such that the RMP hyperpolarizes to approximately −80 mV (Figure 3c). Under these conditions, the upstroke velocity of the subsequently elicited AP accelerated, which indicated an appropriate Nav1.5 expression. For a correct comparison between isolated hSC-CMs versus hSC-CMs in monolayer, all of the reported data were obtained from spontaneous APs, i.e. without current injection.

**Figure 4.**
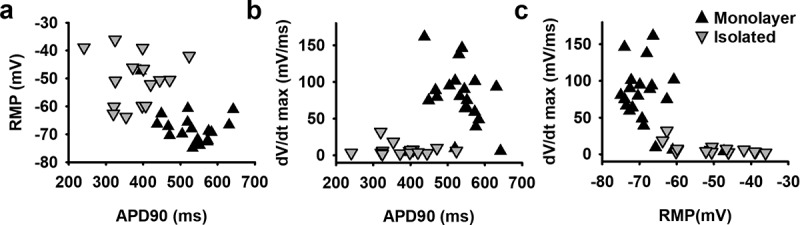
Correlation between RMP, AP duration and upstroke velocity properties in monolayer and isolated hSC-CMs. (a) The RMP of monolayer cells (black triangles) and isolated cells (inverted gray triangles) are plotted against their respective APD90. It is observed that the RMP in monolayer cells is more hyperpolarised compared to the isolated cells and they have a lower variability in APD90 values. Each dot represents the mean of 5 AP´s recorded within one cell. (b) Plot shows the relation between maximum upstroke velocity (mV/ms), obtained by taking the maximum of the first differential of the depolarization phase of the AP, and APD90 (ms). (c) Maximum upstroke velocity is plotted against the respective RMP. Note that when the RMP is more depolarized than −60 mV no adequate upstroke velocity was obtained

By comparing hSC-CM monolayer and isolated cells, it was observed that representative ventricular phenotype APs were mostly from hSC-CM monolayer cells (94%). Most importantly, the RMP of hSC-CMs in monolayers was significantly more hyperpolarized (Δ = −18.8 mV, p-value <0.001) compared to that of isolated hSC-CMs (Table 1). Since the RMP influences sodium current availability at rest, it consequently influences peak potential, AP amplitude and upstroke velocity (mV/ms) [30]. A more depolarized RMP will cause more sodium channels to be inactivated, as the V_1/2_ for inactivation is around −80 mV, such that fewer channels are available to open [31]. In agreement, in monolayers the upstroke velocity of hSC-CMs was significantly faster (approximately a 10-fold, p-value <0.001) compared to isolated cells (Table 1). This correlates well with the reported sodium channel properties of these hSC-CMs whereby almost all sodium channels are inactivated at potentials above −60 mV [31]. A decrease of sodium channel availability is also reflected in peak potential, which in hSC-CM monolayer amounted to 50.4 ± 2.6 mV (n = 20) compared to only 34.7 ± 1.8 mV (n = 16) in isolated cells, consequently resulting in a larger mean AP amplitude in monolayer recordings (Table 1). Accordingly, APD90 of isolated cells was significantly shorter than that of hSC-CM in monolayer (p-value <0.001, Table 1). It needs to be noted again that the recordings were done at room temperature and since APD is inverse temperature-dependent, the obtained APD90 data cannot directly be compared to native CM data at 37°C [32–34]. When the RMP of each cell is plotted against APD90, isolated cells show a marked shorter APD90 than hSC-CMs in monolayer concomitantly with a more depolarized RMP (Figure 4a). Plotting upstroke velocity to RMP, a clear separation between hSC-CM in monolayer and isolated cells is observed whereby cells with an RMP that is more hyperpolarised than −60 mV yielded faster upstroke velocities, especially seen in hSC-CMs of a monolayer (Figure 4c).

**Table 1.** Schematic representation of all main AP waveform parameters analyzed

	Monolayer			n	Isolated			n	p-value
RMP (mV)	−68.1	±	1.4	20	−49.3	±	2.3	16	<0.001
Peak (mV)	50.4	±	2.6	20	34.7	±	1.8	16	<0.001
Amplitude (mV)	118	±	3.2	20	85.3	±	3.1	16	<0.001
MDP (mV)	−73.6	±	0.8	20	−66.5	±	1.5	16	<0.001
APD 90 (ms)	530	±	13.0	20	381	±	17.8	16	<0.001
dV/dt (mV/ms)	77.2	±	9.0	20	7.7	±	2.0	16	<0.001
Rs (GΩ)	2.0	±	0.2	20	2.1	±	0.1	16	0.8
Ri (GΩ)	0.036	±	0.005	20	0.148	±	0.02	16	<0.001
Rs/Ri	74.2	±	10.2	20	18.2	±	1.9	16	<0.001

Values obtained are represented as mean ± S.E.M. and with n = number of cells analyzed. P-value is calculated for each parameter and is significant when <0.05. Note that it is clearly observed that between monolayer and isolated cells all parameters are significantly different except for the seal resistance.

**Figure 5.**
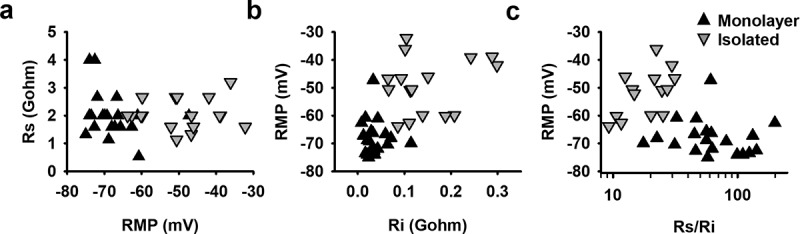
Seal and input resistance can modify RMP recordings from monolayer and isolated cells: (a) The seal resistance Rs of patched hSC-CMs in monolayer (black triangles) and isolated hSC-CMs (gray inverted triangles) is plotted against the RMP. Represented values are the mean of 5 APs recorded from the same cell. (b) RMP was plotted over their respective Ri which visualizes that for isolated cells the RMP is more depolarized when Ri is increased. (c) Plotting the ratio of Ri/Rs over RMP for monolayer and isolated cells clearly states that, mostly, when Rs/Ri ratio is >30 the RMP is more depolarized. Note that the Rs/Ri ratio is plotted as a log scale

The significant difference of approximately −20 mV in RMP observed between hSC-CMs in monolayer and isolated cells is most likely not the result of differences in differentiation state because the cells originated from the same batch of hSC-CMs. However, due to interconnections between cells in a monolayer, the total I_K1_ expression is most likely increased compared to isolated cells (Figure 1), analogous to the gap junction coupling in astrocytes that results in syncytial isopotentiality [35,36]. The formation of a syncytium should also decrease the input resistance (Ri) in hSC-CMs in monolayer compared to the isolated cells. Because the quality of the connection between patch pipette and cell is most likely the same in isolated and monolayer cells, which is reflected in the Rs, the Rs/Ri ratio will differ between isolated cells and cells in monolayer. To evaluate this, we calculated the Ri and Rs from each cell. Although patching cells in monolayer can be more challenging, no difference was observed in the seal quality and the accompanying Rs between monolayer and isolated hSC-CMs (Figure 5a, Table 1). On the other hand, as expected, the Ri was significantly (p-value <0.001) lower in hSC-CM in monolayer compared to isolated cells (Figure 5b, Table 1). The calculated Rs/Ri ratio was significantly lower (p-value = <0.001) in isolated cells (18.2 ± 1.9, n = 16) compared to monolayer hSC-CMs (74.2 ± 10.2, n = 20). By plotting RMP against the Rs/Ri ratio, a clear association was observed between lower Rs/Ri ratios and more depolarized RMPs (Figure 5c).

**Figure 6.**
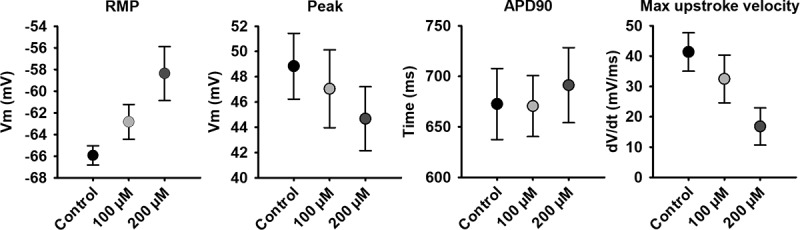
Effect of Cbx on the AP of hSC-CMs in monolayer. The RMP, peak, APD90 and maximal upstroke velocity of the AP from hSC-CMs in monolayer are represented for control condition (normal ECS) and upon 5 minutes wash-in of 100 µM or 200 µM Cbx. Note that these experiments were performed on a different batch of hSC-CMs, explaining the slight differences in values compared to the data in table 1, due to batch-to-batch variability in hSC-CMs

The hyperpolarized RMP of hSC-CMs forming a syncitium is most likely because of an increased I_K1_ expression and an altered ratio between membrane repolaring I_K1_ and aspecific leak currents through Rs. Because the cells are both electrically and structurally connected within the monolayer, we determined if it is the electrical connectivity that yields the hyperpolarized RMP by adding Carbenoxolone (Cbx), a chemical blocker of gap junctions and hemichannels [37,38], while performing the patch clamp experiment. Thus, the AP and RMP of a hSC-CM within a monolayer were monitored for at least 5 minutes upon adding 100 or 200 µM Cbx. In control conditions the hSC-CM displayed a RMP of −65.9 ± 0.9 (n = 12) and upon wash-in of 100 µM and 200 µM Cbx the RMP depolarized to −62.8 ± 1.6 (n = 12) and −58.4 ± 2.5 (n = 12), respectively (Figure 6). Although only the change in RMP upon 200 µM Cbx addition was significantly different from control (p-value = 0.007), there was a Cbx concentration-dependent effect. Due to the depolarization of the RMP, the upstroke velocity (dV/dtmax) slowed down and the peak potential decreased (Figure 6). The APD90 did not alter significantly, suggesting that other ionic currents were not substantially affected by Cbx.

It has to be noted that I_K1_ currents could also be balanced out by other leak currents, not originating from the Rs, such as those from gap junction hemichannels (Figure 1), which are well expressed in hSC-CM[39]. Although hemichannels should be closed, they could open during depolarization [40]or by chemical stimuli such as a change in pH or intracellular Ca^2+^ concentration. The number of hemichannels in isolated cells will be higher than in monolayer cells because hemichannels from neighboring cells form gap junction channels. To evaluate the contribution of current through hemichannels in depolarizing the RMP of isolated hSC-CMs, we evaluated the effect of hemichannel inhibition with Gap-26 and Gap-27 peptides [23,24,41]. If hemichannels produce a significant leak current, the RMP should be more hyperpolarized after extracellular application of these peptides. Measuring the RMP of isolated hSC-CMs after 3 hours incubation and presence of the peptides while performing the patch clamp experiments, no significant difference in RMP was observed (Table 2). Upstroke velocity was faster compared to the isolated cells in the previous experiments, as in these experiments all parameters were calculated from induced APs.

**Table 2.** AP properties of isolated hSC-CMs in control condition and upon Gap-26/Gap-27 exposure

	Control			n	Gap-26			n	p-value
RMP (mV)	−44.2	±	5.4	9	−50.5	±	2.2	8	0.32
Peak (mV)	44.5	±	2.5	9	52.8	±	5.1	8	0.15
Amplitude (mV)	93.0	±	5.65	9	108	±	5.99	8	0.08
MDP (mV)	−48.5	±	5.2	9	−55.9	±	2.2	8	0.23
APD 90 (ms)	366	±	39.4	9	494	±	64.5	8	0.10
dV/dt (mV/ms)	50.3	±	6.5	9	71.9	±	7.3	8	0.04
	Control			n	Gap-27			n	p-value
RMP (mV)	−41.6	±	7.7	5	−53.0	±	4.0	4	0.27
Peak (mV)	52.9	±	6.5	5	46.0	±	8.8	4	0.54
Amplitude (mV)	97.3	±	11.2	5	102	±	9.62	4	0.76
MDP (mV)	−45.1	±	7.1	5	−56.1	±	3.6	4	0.25
APD 90 (ms)	369	±	87.7	5	449	±	38.4	4	0.47
dV/dt (mV/ms)	72.4	±	11.9	5	49.7	±	14.6	4	0.26

Values obtained are represented as mean ± S.E.M. and with n = number of cells analyzed. P-values were calculated as described in the methods section.

**Figure 7.**
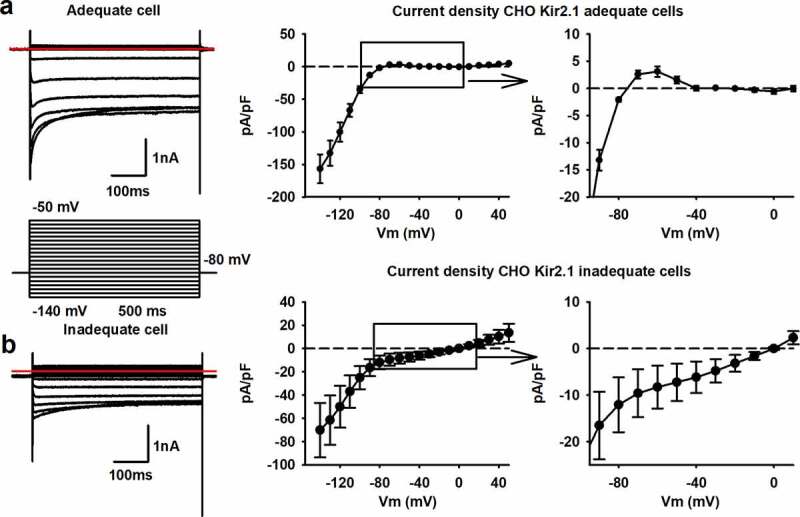
Rs and Ri influence the reversal potential of the Kir2.1 channel. (a) Representative current traces of an adequate CHO cell that expresses sufficient Kir2.1. The red line indicates the zero level. The voltage pulse protocol to elicit the currents is shown below. On the right is the current density (pA/pF) plotted to their respective potentials, generating an IV plot, with a magnification of the positive current around −80 mV. Note that the current density was determined without performing a leak subtraction on the recording. (b) Example traces of an inadequate CHO cell, using the same voltage clamp protocol, expressing low levels of Kir2.1 with the red line indicating the zero level. On the right is the current density (pA/pF) plotted to their respective potentials with a magnification of the negative current until 0 mV

To further explore that the ratio of Rs versus I_K1_ expression influences the RMP, we transiently expressed the I_K1_ current (Kir2.1 channel) in CHO cells. Since transient transfection efficiencies are variable, some cells will express more I_K1_ than others. The higher the level of I_K1_ expression, the lower the Ri should be (when determined around −80 mV). I_K1_ currents were measured in ruptured patch configuration, applying a step-pulse protocol ranging from −140 mV to +50 mV (Figure 7). The Kir2.1 channel is constructed by tetramerization of alpha subunits that consist out of two transmembrane domains[42]. As such, no voltage-sensing domain is present and Kir2.1 is a non-voltage gated K^+^ channel that displays inward rectification due to intracellular Mg^2+^ block at depolarizing potentials. Depolarized potentials, above the K^+^ reversal potential (i.e. around −80 mV with the solutions used), induce outward potassium currents but intracellular Mg^2+^  will start to block the Kir2.1 channel and reduce the I_K1_ amplitude (Figure 7a). In cells, the outward current will repolarize the cell to −80 mV resulting in the stabilization of the RMP around this value when I_K1_ expression is sufficient to overcome background leak (reviewed in [42]). Our non-transfected CHO cells have an RMP of around −1.9 ± 1.3 mV (n = 6), which is in line with reported data [43]. Almost all cells had an Rs higher than the recommended 1 GΩ and the small variability in Rs will have no major effect on the Rs/Ri ratio. Analysis of cells with a high expression of I_K1_, peak pA/pF at −140 mV of −157 ± 22 pA/pF (n = 8), resulted in a Ri of 0.09 ± 0.03 GΩ. The RMP of these cells was close to the reversal potential of K^+^ with the solutions used and amounted to −78 ± 1 mV, respectively. The high expression of I_K1_ is therefore linked to a lower Ri as more Kir2.1 channels are present. The RMP value was selected as the best cutoff value for group division of the cells since it influences multiple factors of the AP waveform in hSC-CMs. Cells were divided in an “adequate group” with RMP values around −80 mV and an “inadequate group” with RMP values around 0 mV. Accordingly, cells having an adequate RMP also displayed a higher Rs/Ri ratio compared to cells with inadequate RMP values (Table 3).

**Table 3.** RMP, current density, Rs and Ri values of I_K1_ expressing CHO cells divided in an adequate and inadequate group

	Adequate cells			n	Inadequate cells			n	p-value
RMP (mV)	−78.15	±	1.02	8	−1.89	±	1.25	6	<0.001
Peak pA/pF at −140 mV	−157	±	22.1	8	−70.1	±	23.4	8	0.018
Peak pA/pF at −60 mV	3.09	±	0.96	8	−8.29	±	4.60	8	0.03
Rs (GΩ)	3.04	±	0.48	8	1.31	±	0.21	6	0.021
Ri (GΩ)	0.09	±	0.03	8	6.35	±	2.01	6	0.004
Rs/Ri	737	±	431	8	0.40	±	0.09	6	<0.001

Values are represented as mean ± S.E.M. and n = number of cells analyzed. P-value <0.05 indicates statistically significant difference.

To elucidate the effect of the leak currents through the seal on I_K1_ recordings, the I_K1_ current density-voltage (IV) plots were generated without performing leak subtraction. In qualitative cells with a low Ri and high Rs (Rs/Ri ratio >5), a positive current flows at membrane potentials above the reversal potential of K^+^, which is then sterically blocked by Mg^2+^ upon stronger depolarizations. Consequently, a positive “hump” is seen between −80 mV and −40 mV (Figure 7a). This is not observed in the IV plots of cells with a high Ri, suggesting an insufficient expression of the Kir2.1 channel and therefore a low I_K1_ current (Figure 7b). While the Rs is high, the I_K1_ amplitude is not sufficient to surpass the leak current and the IV plot does not cross the zero current level at −80 mV. Accordingly, in inadequately patched cells with enough I_K1_ expression, thus a low Ri but also a low Rs due to a bad seal, the IV plot crosses the zero current level at more depolarized potentials than −80 mV (i.e. the K^+^ reversal potential with the solutions used), in some cases even at 0 mV (the reversal potential of ion nonselective leak currents). Interestingly, when the seal was of sufficient in quality, a very high expression of Kir2.1 appeared not to be necessary as a mean I_K1_ current density of 3.1 ± 1 pA/pF (n = 8) at −60 mV was sufficient to generate an IV plot with a reversal potential of −78 ± 1 mV (n = 8).

## DISCUSSION

Most reports on the RMP and AP waveform of hSC-CMs and hiPSC-CMs present data from isolated cells that were dissociated from their monolayers or 3D structured cultures 4 to 10 days prior to the measurements [13,44–47]. On the other hand, native CMs are mostly recorded immediately after dissociation, as longer culturing of these cells leads to more depolarized RMP values and larger AP waveform variability [48]. The main and most straightforward explanation postulated for the depolarized RMP in hiPSC-CMs and hSC-CMs is a reduced expression of Kir2.1, resulting in a lower I_K1_ current and losing the ability to balance the RMP around −85 mV (close to the reversal potential of K^+^ in physiological conditions) [49–51]. Using the same cells and applying the same perforated patch clamp technique, our data show that the RMP of an hSC-CM cell that is interconnected with neighboring cells is approximately −20 mV more hyperpolarized compared to isolated cells. Accordingly, chemical isolation of the cells within the monolayer by application of Cbx depolarized the RMP (Figure 6). Since Cbx blocks the gap junctions [37,38] without affecting the structural connectivity substantially, the hyperpolarized RMP of hSC-CMs in a monolayer is because of electrical coupling and the formation of a syncytium. Since gap junctions are absent in isolated cells, the hemichannel population is most likely increased. Pre-treatment of the isolated hSC-CMs with hemichannel blocking Gap peptides did result in a small hyperpolarization of the RMP (Table 2). However, the change was not sufficient to be significant, indicating that the contribution of hemichannels in depolarizing the RMP of isolated cells is minor and in line with a very low open hemichannel probability at rest.

Recent studies have suggested that, while I_K1_ has a lower expression, there could also be a technical factor that contributes to the depolarized RMP of hSC/hiPSC-CM [16,17]. To minimize the contribution of nonspecific ionic leak current through the seal during a patch clamp experiment, it has been reported that the Rs should be at least 5 times larger than the Ri [17]. When comparing AP waveforms from isolated and monolayer hSC-CMs, this Rs/Ri ratio indeed had an influence on RMP whereby the Ri appeared the main variable between both models. When calculating the Rs/Ri ratio, isolated cells had on average a lower Rs/Ri ratio compared to hSC-CM monolayer cells. It has to be noted that the isolated hSC-CM cells also achieved a ratio above 5. It is possible that for hSC-CM cells the ratio has to be larger than 5, as was reported from an *in silico* model [17].

The significant difference in RMP between an hSC-CM in a monolayer and an isolated hSC-CM is most likely due to the interconnection of the cells in a monolayer, which results in a lower Ri and increased I_K1_ expression. The increase in I_K1_ expression can be ascribed to either an increase in the amount of Kir2.1 channels or/and the conductivity of expressed Kir2.1 is increased because of the cell-cell connections. The latter is based on the observation that Kir2.1 expression localizes at the intercalated discs and transverse tubule system [52]. On top, Kir2.1 is reported to form macro complexes with Nav1.5 channels that localizes at the same position, which in turn increases the current density of both channels [53,54]. By creating cell-cell connections in monolayers, while not completely resembling the physiological environment, these channel complexes could be expressed at more favorable locations and consequently increasing I_K1_. T-tubules are, however, not well developed in hSC-CMs models while being an essential structure in mature native cardiomyocytes [55–57]. Thus, allowing hSC-CMs to form a syncytium results in a more hyperpolarized RMP compared to isolated cells, but with an average value of −68.1 ± 1.4 mV (n = 21) it remains approximately 15 mV more depolarized than native CMs, which can be attributed to immaturity and lower Kir2.1 expression or due to morphological differences that affect the functional I_K1_ expression. There are maturation studies for hiPSC-CMs reporting RMP values around −85 mV, including 3D culturing methods [10,13,14,16,58,59].

Transient transfection of CHO cells with the Kir2.1 channel supported that Ri is associated with the expression level of this channel and that the Rs/Ri ratio affects the current density versus voltage (IV) plots (Figure 7). When the Rs/Ri ratio was inadequate (e.g. below the theoretically required value of 5 [17]), the RMP of the cell did not reach the K^+^ reversal potential, which is set by the Kir2.1 channel, and the nonspecific leak current through the seal depolarized the RMP in these cells. Thus, when the Kir2.1 expression is low, the amplitude of the K^+^ current in the voltage range between −80 mV and −50 mV is insufficient to counter the leak current pulling the normally positive part of the IV plot to negative current values (Figure 7b). If this occurs during patch clamp measurements the recorded RMP will depolarize, resulting in an underestimation of the real RMP.

In hSC-CMs in monolayer the RMP seems hyperpolarized enough to induce a fast upstroke of the AP waveform and AP properties of hSC-CMs are more comparable to native human ventricular CMs than that of isolated cells supporting their usability in pharmacological screening assays. Combined with Nav1.5 current data from previous research, the RMP needs to be at least lower than −60 mV as otherwise most Nav1.5 channels will be inactivated and consequently not available for activation [30,31]. In our patch clamp recordings, we only observed a fast upstroke velocity when the RMP was more hyperpolarized than −60 mV. hiPSC-CMs and hSC-CMs are becoming more widely used in high-throughput systems such as automated patch clamp platforms, imaging systems using Ca^2+^ /voltage dyes, microelectrode array (MEA) recordings, and/or impedance measurements whereby the latter three techniques use monolayers [4,60,61]. Therefore, it has to be noted that due to potential batch-to-batch variability in differentiation status of hiPSC-CMs and hSC-CMS, every batch should be evaluated before use. While MEA systems cannot record the RMP directly, the upstroke velocity can be determined and this parameter is related to the RMP as we show here (Figure 4c). Variability in RMP between differentiated hSC-CM and hiPSC-CM batches can underlie variability in drug effects. For example, phenytoin, which is a low-risk blocker at its clinical dose, has been reported to result in a beat stop of the hSC-CM monolayer in approximately 50% of the experiments, probably caused by a more depolarized RMP [4].

In conclusion, while the expression of the Kir2.1 channel might still be lower in hSC-CMs compared to native CMs, due to the interconnection of hSC-CMs in monolayer the amount of I_K1_ expression seems sufficient to maintain an almost physiological RMP. This finding can most likely be extended to hiPSC-CMs as well and more physiological representative ventricular type of APs can be generated by recording from hSC-CMs and hiPSC-CMs in a monolayer forming a syncytium.

## Data Availability

The generated datasets of this study are available on request.
